# Determination of the Concentration of Heavy Metals in Artisanal Cheeses Produced in the Mexican States of Tabasco and Chiapas

**DOI:** 10.3390/molecules28237907

**Published:** 2023-12-02

**Authors:** Francisco Anguebes-Franseschi, Mohamed Abatal, Claudia Alejandra Ucán, Alejandro Ruiz Marín, Francisco Tamayo-Ordoñez, Yunuen Canedo-López, Luis Perez-Reda, Siprian Damás-Damas

**Affiliations:** 1 Facultad de Química, Universidad Autónoma del Carmen, Calle 56 No. 4 Esq. Av. Concordia, Col. Benito Juárez, Ciudad del Carmen 24180, Campeche, Mexico; caguilar@pampano.unacar.mx (C.A.U.); armarin@delfin.unacar.mx (A.R.M.); ftamayo@pampano.unacar.mx (F.T.-O.); ycanedo@pampano.unacar.mx (Y.C.-L.); lperez@pampano.unacar.mx (L.P.-R.); sdamas@pampano.unacar.mx (S.D.-D.); 2 Facultad de Ingeniería, Universidad Autónoma del Carmen, Campus III, Avenida Central S/N, Esq. Con Fracc. Mundo Maya, Ciudad del Carmen 24115, Campeche, Mexico; mabatal@pampano.unacar.mx

**Keywords:** food analysis, health, physical chemistry

## Abstract

Cheese consumption provides humans with minerals, proteins, carbohydrates, and vitamins. In Mexico, several cheese varieties are produced, each with its texture, scent, and flavor. The artisanal cheeses made in the states of Tabasco and Chiapas—including, among others, the varieties named *crema* (cream), *doble crema* (double cream), *oaxaca*, *panela*, *fresco*, *bola*, *poro*, *cotija*, and *asadero*—have a high demand in the domestic and foreign markets. The intensification of anthropic activity in these states causes an increased emission to the environment of contaminants like heavy metals, which could reach human foodstuffs through the food chains. In particular, heavy metal contents in cheeses consumed daily by these states’ local populations might represent a public health risk. Because of that, our objectives in this work were to determine the concentrations of lead, cadmium, nickel, copper, zinc, and iron in artisanal cheeses produced in the states of Tabasco and Chiapas and to determine the values of the hazard quotient (*HQ*), total hazard quotient (*THQ*), and cancer risk total (*CRT*) for adult and young men and women. The results of our analyses of cheese samples from the states of Tabasco and Chiapas showed that the average concentrations (mg kg^−1^) of cadmium (0.0023 ± 0.002, 0.0023 ± 0.002 mg kg^−1^, respectively, for each state), lead (0.0047 ± 0.00, 0.0051 ± 0.002), nickel (0.0039 ± 0.0046, 0.0031 ± 0.0039), copper (0.0199 ± 0.021, 0.0202 ± 0.022), zinc (0.1611 ± 0.18, 0.194 ± 0.21), and iron (61.84 ± 4.23, 65.76 ± 6.61 mg kg^−1^), the first three values lower than the limits established by the FAO/WHO and *Codex Alimentarius*. The value of *THQ* that we obtained was less than one, and that of *CRT* was within the limits established by the US-EPA, which means that the consumption of artisanal cheeses from Tabasco and Chiapas by humans does not imply a risk of disease or cancer.

## 1. Introduction

The large variety of cheeses produced in different geographic regions represents healthy foodstuffs for human consumption because they contain high concentrations of proteins, fats, vitamins, and minerals—including macro (Na, K, Ca) and micro (Zn, Cu, Fe) elements, each having their own organoleptic and physicochemical properties [1]. In each geographic region, the natural grasslands, fodders, and grains fed to cattle might represent sources of contaminants of cheeses. In particular, heavy metals bioaccumulate in the muscles, organs, and bones of animals, thus contaminating dairy products like milk, whey, cream, butter, and cheeses whose consumption might be a risk to human health. The intake of foodstuffs containing heavy metals has become a public health issue because of their carcinogenic, mutagenic, and cytotoxic effects [2,3], because of which the Food and Agriculture Organization (FAO)/World Health Organization (WHO) [4], *Codex Stan Alimentarius* [5], EFSA (European Food Safety Authority) [6], and Official Mexican Standard (NOM-243-SSA1-2010) have established strict regulations of the maximum permissible concentrations of heavy metals in foodstuffs [7]. The risk for health of the intake of heavy metals is assessed using the hazard quotient (*HQ*), total hazard quotient (*THQ*), and cancer risk total (*CRT*) indexes established by the United States Environmental Protection Agency (US-EPA) [8], which are of common use globally [9].

Mexico produces a large variety of industrial and artisanal cheeses, using nearly 25% of the country’s total milk production. The nearly 40 varieties of artisanal cheeses made in Mexico account for approximately 70% of the total domestic cheese production, estimated at nearly 400 thousand tons per year, and the per capita consumption in the country ranges from 2.1 to 6 kg yr^−1^ [10]. The varieties of artisanal cheeses produced in the Mexican states of Tabasco and Chiapas include *queso fresco* (fresh cheese), *queso crema* (cream cheese), *queso doble crema* (double cream cheese), *panela* (Mexican cottage cheese), *queso oaxaca* (Mexican string cheese), *queso cotija* (Mexican mild feta cheese or, when aged, Mexican Parmesan cheese), *queso asadero* (grillable cheese), and *queso de bola* (ball-shaped cheese or Mexican Edam cheese), and in smaller amounts, mozzarella and provolone cheeses of the above-mentioned artisanal cheese varieties, oaxaca, fresco, panela, cream, and double cream have high demand in the local, regional, and global markets. There has been a recent increase in heavy metal environmental contamination in Tabasco and Chiapas during the past years due to some anthropic activities—mostly, the petroleum industry, technicized agriculture, and internal combustion engines, because of which, in this work, our objectives were to determine the concentrations of cadmium (Cd), lead (Pb), nickel (Ni), copper (Cu), zinc (Zn), and iron (Fe) in the following eight varieties: (a) double cream, (b) cream or soup, (c) oaxaca, (d) panela, (e) cotija, (f) fresco, (g) mozzarella, (h) provolone of artisanal cheeses made in the states of Tabasco and Chiapas, and the values of the *HQ*, *THQ*, and *CRT* indexes for these cheeses.

## 2. Results and Discussion

Table 1 and Table 2 show the average, standard deviation, minimum value, and maximum value of the concentrations of heavy metals in the artisanal cheese samples from Tabasco and Chiapas, respectively. In the following sections, we discuss our results for each analyzed heavy metal.

### 2.1. Statistical Analysis 

In this study, ANOVA with Tukey and paired Student’s *t*-test were performed using the InfoStat/L version 2020 software to know if the differences between the moisture content and the concentrations of Pb, Cd, Ni, Cu, Zn, and Fe observed in the 88 cheese samples were significant (Table 3, Table 4 and Table 5).

### 2.2. Lead

The International Programme of Chemical Safety (IPCS) [11] and the International Agency for Research on Cancer (IARC) [12] classify lead as a probable human carcinogen (2A). The high toxicity of lead might cause diseases and, at high concentrations, several types of cancer [13,14]. Our results showed that the concentration of lead in the 88 artisanal cheese samples we analyzed was below the 0.02 mg kg^−1^ and 2 mg kg^−1^ maximum limits for dairy products for human consumption established by the European Commission EC. (2001) [15] and FAO/WHO [4], respectively. The 44 analyzed samples from Tabasco had an average lead concentration of 0.0041, with a minimum of 0.0002 and a maximum of 0.0125 mg kg^−1^. By artisanal cheese variety, the average, minimum, and maximum values of lead concentration in mg kg^−1^ were as follows: 0.0056, 0.0004, and 0.0125 for cream or soup and double crema; 0.0045, 0,0028, and 0.0084 for oaxaca; 0.0037, 0.0005, and 0.0068 for fresco; and 0.0041, 0.0031, and 0.0047 for panela. For the 44 samples from Chiapas, we found average, minimum, and maximum values of lead concentration in mg kg^−1^ of 0.0051, 0.0003, and 0.0089. For the latter state’s artisanal cheese varieties, the average, minimum, and maximum values of lead concentration in mg kg^−1^ were as follows: 0.0062, 0.0029, and 0.0089 for crema; 0.0054, 0.0039, and 0.0088 for cotija; 0.0044, 0.0003, and 0.0083 for oaxaca; and 0.0028, 0.0005, and 0.0078 for panela. 

**Figure 1:**
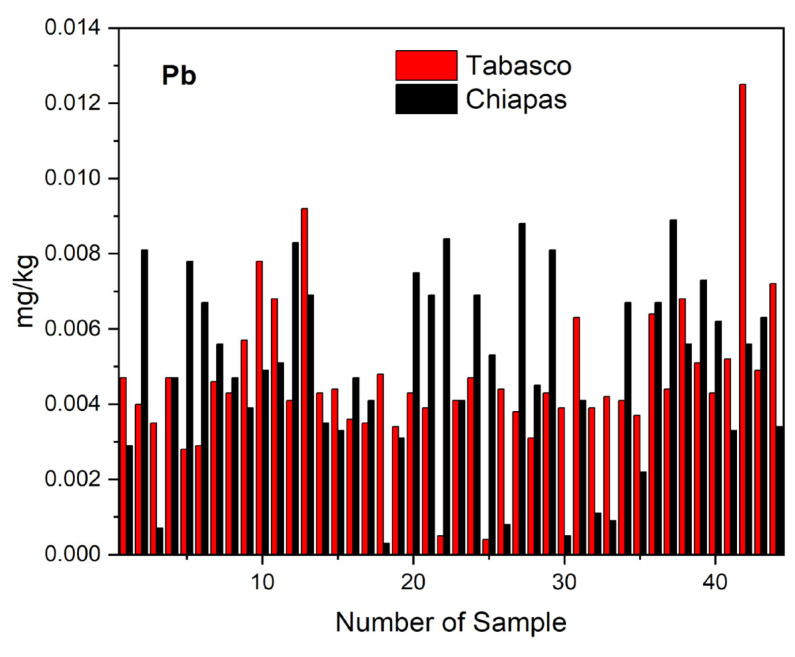
Distribution of lead in the different varieties of artisanal from Tabasco and Chiapas.

Figure 1 shows the distribution of the lead concentration values that we observed in the samples of artisanal cheese varieties crema, oaxaca, panela, cotija, and fresco we collected in the states of Tabasco and Chiapas. The maximum lead concentration of 0.0125 mg kg^−1^ that we observed corresponded to a sample of crema artisanal cheese from the municipality of Huimanguillo, Tabasco could have been due to pollution during the manufacturing process [16]. In general, the varieties cotija and crema had higher lead concentrations than the varieties oaxaca, fresh, and panela, which could be explained by the former varieties having a less content of water by weight (38.699% and 45.264%, respectively) than the latter (59.546, 60.799, and 61.325%) see Table 3. In addition, the Tukey analysis showed that there are significant differences in moisture content between cotija and cream cheeses with respect to panela, oaxaca, and fresh cheeses (Table 4). The results of the statistical analysis of the lead concentrations in the artisanal cheeses from the state of Tabasco and Chiapas gave a value of TC = −0.661 for the Student’s *t*-test for paired data, which is within the range of critical value (CV between −2.672 and +2.672), indicating that there are no significant differences in the lead content in the cheese samples, which confirms the results from the Tukey test (Table 5a), which could mean that in both processes the contamination sources were the same. 

In a study of fresco cheese from the Mexican state of Puebla, by Benítez found an average lead concentration of 2.96 mg kg^−1^ [16]. Castro analyzed the lead concentration values in artisanal cheeses made in Santa Ana Xalmimilulco, Puebla, reporting averages of 0.11 ± 0.04 mg kg^−1^ for ranchero and 0.05 ± 0.03 mg kg^−1^ for Oaxaca cheese varieties, the authors attributed the presence of lead in the cheeses to the alfalfa fed to cattle being irrigation with polluted water from the Atoyac River [17]. In studies made in Turkey, Yüzbaşi found lead concentrations between 0.0364 and 0.251 mg kg^−1^ in the artisanal Kasar cheese samples from Ankara [18], and Bakircioglu reported lead concentrations ranging from 0.60 ± 0.17 and 0.48 ± 17 mg kg^−1^ for cream and white cheese made in Edirne [19]. Moreno analyzed 57 varieties of cheese from different regions of Spain and found that their lead contents were between 5.0 ± 0.01 and 110.0 ± 8.2 µg kg^−1^ [1]; while in Italy, Lante reported a lead concentration of 0.06 mg kg^−1^ in crescenza and squacquerone cheeses [20]. 

### 2.3. Cadmium

The IARC and IPCS [11,12] determined that the ingestion of even a small amount of cadmium is highly toxic for humans; therefore, determining its presence in food is essential. The results of our determination of the content of Cd in cheese samples from Tabasco show average concentrations between 0.0023 ± 0.0025 and 0.0056 mg kg^−1^. By cheese variety, the average, minimum, and maximum values of cadmium concentration in mg kg^−1^ were as follows: 0.0045, 0.0019, and 0.0156 for crema; 0.0009, 0.00, and 0.0031 for oaxaca; 0.0011, 0.0002, and 0.0037 for fresco; and 0.0019, 0.0001, and 0.0075 for panela. For the analyzed cheese samples from Chiapas, we found an average Cd concentration of 0.0023 ± 0.0025 with a minimum of 0.00 and a maximum of 0.0055 mg kg^−1^. By cheese variety, the average, minimum, and maximum values of cadmium concentration in mg kg^−1^ were as follows: 0.0043, 0.0029, and 0.0077 for crema; 0.0042, 0.0032, and 0.0062 for cotija; 0.0019, 0.00, and 0.0046 for oaxaca; and 0.0014, 0.0002, and 0.0034 for panela. 

**Figure 2:**
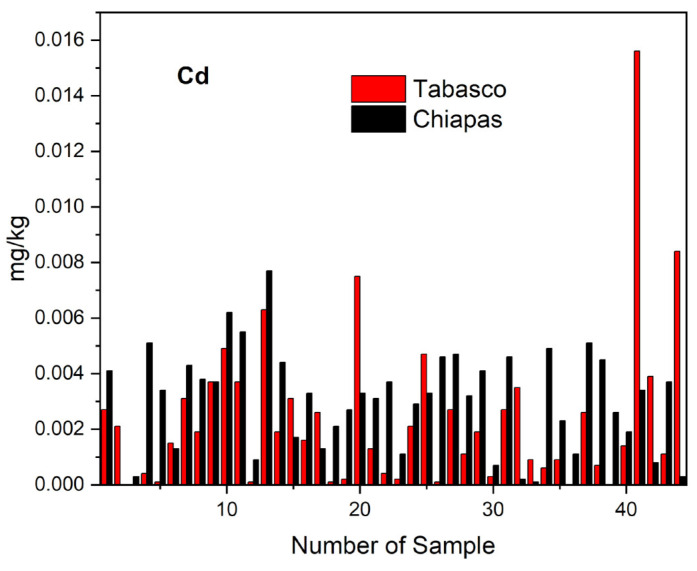
Distribution of cadmium in the different varieties of artisanal cheeses from Tabasco and Chiapas.

The distribution of the cadmium concentrations we found in the crema, oaxaca, panela, cotija, and fresco cheese samples from Tabasco and Chiapas shows that the highest Cd concentrations correspond to crema and cotija cheeses, and the lowest Cd concentrations, to the panela, oaxaca, and fresco samples (Figure 2). The (Table 3) latter result may have been due to the lower water content of the crema (<45.264% by weight) and cotija samples (<38.699%) relative to that in the panela, oaxaca, and fresco (>59%). The results obtained from the statistical analysis of cadmium concentrations in Tabasco and Chiapas cheeses showed that there are no significant differences between cheese samples since the value of the paired t-Student test (TC = −1.318) is within the range of critical value (VC between −2.672 and +2.672). Furthermore, the results of the Tukey’s test confirmed this result (Table 5b), which suggests that the possible sources of cadmium pollution were similar in both states. We found that the cadmium concentrations in all the samples we analyzed were under the maximum values established by the Codex Alimentarius 0.05 mg kg^−1^ [5] and the 1.0 mg kg^−1^ FAO/WHO [4]. 

In a study made in the Mexican state of Puebla, Castro found the presence of cadmium in urine, blood, and milk of Holstein cattle in the Santa Ana Xalmimilulco region in the municipality of Huejotzingo and determined its concentration in milk samples to be 0.54 mg kg^−1^, which the authors attributed to the cattle’s ingestion of alfalfa containing heavy metals [21]. In their study of the heavy metal content in samples of fresco cheeses sold in markets in the city of Puebla, Benítez found heavy metals, including an average Cd concentration of 0.13 mg kg^−1^ [16]. In Egypt, Meshref reported an average cadmium concentration of 0.09 mg kg^−1^ in Kareish in the Beni-Suef region [22]. Elbarbary and Hamouda found 0.24 mg kg^−1^ of cadmium in feta cheese samples [23]. In Turkey, Çetinkaya reported an average cadmium concentration of 0.028 mg kg^−1^ in cami bogazi cheese samples from the Trabzon region [24]. The concentrations of cadmium in cheeses reported in the literature that we found were higher than those we observed in our study. 

### 2.4. Nickel

The United States Department of Health and Human Services (DHHS) determined that metallic nickel and some nickel compounds are carcinogens, and the ingestion of high Ni concentrations can be hazardous to human health [25,26]. The FAO and European Commission (EC) have not established the permitted maximum intake limit of nickel for humans [4,15]; however, the Integrated Risk Information System of the United States Environmental Protection Agency (US-EPA-IRIS) established a 300 µg/kg limit [14,27], and the WHO established a tolerable daily intake of 11 μg kg^−1^ of body weight for children and 12 μg kg^−1^ of body weight for adults [28]. 

**Figure 3:**
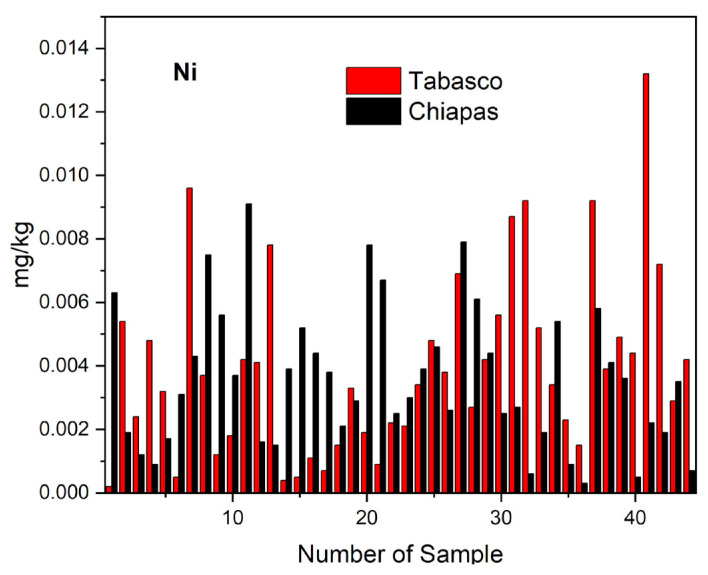
Distribution of nickel in the different varieties of artisanal cheeses from Tabasco and Chiapas.

The results of our analysis of artisanal cheese samples from Tabasco showed an average nickel concentration of 0.0039 ± 0.0046 mg kg^−1^, a maximum value of 0.0132 mg kg^−1^, and a minimum of 0.0002 mg kg^−1^. By cheese variety, the average, maximum and minimum values in mg kg^−1^ were as follows: 0.0059, 0.0002, and 0.0132 for crema; 0.0029, 0.0005, and 0.0049 for oaxaca; 0.0031, 0.0021, and 0.0042 for fresco; and 0.0029, 0.0004, and 0.0056 for panela. For the cheese samples from Chiapas, we found an average Ni concentration of 0.0031 ± 0.0039 mg kg^−1^ with a minimum of 0.0003 mg kg^−1^ and a maximum of 0.0091 mg kg^−1^. For the same state by variety, we found average, minimum, and maximum Ni concentrations in mg kg^−1^ of 0.0050, 0.0015, and 0.0091 for crema; 0.0052, 0.0027, and 0.0079 for cotija; 0.0031, 0.0016, and 0.0052 for oaxaca; and 0.0017, 0.0006, and 0.0031 for panela. The distribution of nickel concentrations in cheese samples we analyzed from Tabasco and Chiapas (Figure 3) shows that matured cheeses with lower humidity content like crema and cotija have higher concentrations of nickel than the more humidity varieties like oaxaca, panela, and fresco (Table 3 and Table 4). The statistical analysis of the nickel concentrations in the samples yielded a value of TC = +0.698, and Tukey’s test indicated no significant differences (Table 5c), again showing similar contamination sources in both geographic areas. 

Our results compare well with those of the analyses of Ni concentrations made by Castro in samples of oaxaca (0.003 mg kg^−1^) and ranchero (0.01 mg kg^−1^) artisanal cheeses from the Mexican state of Puebla [17]. However, the values of nickel concentration in artisanal cheeses reported by Nöel for French samples (0.409 mg kg^−1^) [29], and by Olujimi for milk (7.70 mg kg^−1^) and cheese (8.33 mg kg^−1^) samples from the Nigerian Ogun and Oyo states are much higher than those reported for Mexico [30]; these authors attributed the contamination with nickel of milk and cheese to oil industry activities and the use of agrochemical inputs.

### 2.5. Sources of Lead, Cadmium, and Nickel in Artisanal Cheeses from Tabasco and Chiapas

The presence of lead, cadmium, and nickel in the artisanal cheeses made in Tabasco and Chiapas might come from the natural and anthropic sources described below:

- Oil industry. Large oil production facilities in Tabasco include Samaria, Jujo, El Golpe, Delta del Grijalva, Bellota, Ogarrio, Cinco Presidentes, and Cuenca de Macuspana, and in Chiapas, Cactus. Southeastern México has 99 oil fields with 935 active wells and 2360 km of oil ducts connecting wells and oil fields with pumping stations and oil processing centers [31]. Manríquez determined heavy metals were present in the Mexican crude oils Maya, Istmo, and Olmeca, reporting concentrations between 8 and 277 mg L^−1^ of vanadium and from 2.5 to 52.0 mg L^−1^ of lead [32]. Siebe reported the presence of lead (0.01–0.1 mg L^−1^), chromium (2.0 mg L^−1^), zinc (0.1–2.5 to 0.2 mg L^−1^), cadmium (0.02–0.04 mg L^−1^), and copper (0.2–0.4 mg L^−1^) [33]. In the Cinco Presidentes oil field well in Tabasco, Fridler analyzed heavy metal concentrations average in groundwater reported for Nickel 9 ug L^−1^, zinc 38 ug L^−1^, copper 16 ug L^−1^, cadmium 0.5 ug L^−1^, and lead 8 ug L^−1^; in sediments, nickel (49–92 mg kg^−1^), chromium (73–138 mg kg^−1^), and lead (8–95 mg kg^−1^) [31]. Villanueva and Botello determined the content of heavy metals in the sediments of the Laguna el Yucateco in the state of Tabasco, and in the muscles of the inhabiting aquatic organisms [34], reporting between 14.27 and 329.24 µg g^−1^ of lead, 0.76–5.52 µg g^−1^ of cadmium, and 44.61 ± 5.38 µg g^−1^ of nickel in sediments, and in the muscle of several edible species, 0.33–4.30 mg kg^−1^ of cadmium, 0.19–15.68 mg kg^−1^ of lead, and 0.1–8.75 mg kg^−1^, of nickel—the latter concentrations being above the maximum limits established in the FAO/WHO and Codex Alimentarius [4,5]. Therefore, the presence of heavy metals is an ecological risk factor because they can move from soils to groundwater, and from there, to aquatic ecosystems where edible species bioaccumulate them;
- Agriculture. In the state of Tabasco, nearly 239,904 ha are used for food production using technicized agriculture [35,36,37,38,39,40], and Chiapas are cultivated as follows: white maize, sorghum, African palm, bean, cacao, and chili are sown in nearly 14,000 ha in the municipalities of Catazajá, Palenque, Reforma, and La Libertad reported by CONACYT and CentroGeo [41,42]. To enhance the yields of crops, large amounts of chemical fertilizers like urea, sulfates, phosphate, and potassium superphosphate are added to the soil, which may be sources of heavy metal pollution. In this regard, Atafar found lead (from 3.32 to 4.28 mg kg^−1^) and cadmium (0.02–1.12 mg kg^−1^) in potassium sulfate, ammonium sulfate, and potassium nitrate [43]. Additionally, Gupta reported concentrations of 0.03 mg kg^−1^ of cadmium and 1.0 mg kg^−1^ of copper in urea, and in potassium superphosphate, 12.2 mg kg^−1^ of cadmium, 60 mg kg^−1^ of zinc, and 22.5 mg kg^−1^ of copper [44]. In phosphate fertilizers, Dissanayake reported the presence of 0.04–65 mg kg^−1^ of cadmium, 1–20 mg kg^−1^ of lead, 11–71 mg kg^−1^ of nickel, 4–130 mg kg^−1^ of copper, and 6–500 mg kg^−1^ of zinc [45];
- Urban areas and roads. The large towns and terrestrial communication networks in the regions of Tabasco and Chiapas where artisanal cheese samples were collected are possible sources of heavy metal pollution. Internal combustion engines used in vehicles and the oil industry might emit gasses containing high amounts of heavy metals [46]. The solid microparticles generated during gasoline and diesel combustion travel long distances dispersing heavy metals and are deposited by gravity on croplands and grazelands [47]; besides, Akpoveta and Osakwe reported contents of 0.24 ppm of lead, 1.68 ppm of cadmium, 1.74 ppm of copper, and 1.43 ppm of zinc in gasolines, and of 1.01 ppm of lead, 1.50 ppm of cadmium, 1.77 ppm of copper, and 2.87 ppm of nickel in diesel [48];
- Processing tools and inputs. The use of metallic utensils and the addition of salt during the manufacturing of artisanal cheeses are possible sources of heavy metal pollution [49];
- Volcanic emissions. The eruption of the Chichonal volcano in 1982 emitted large quantities of ashes over Chiapas, Tabasco, Campeche, and southern Veracruz [50]. In this regard, Rincón analyzed the sediments from the Chichonal volcano finding 3.26–7.06 mg kg^−1^ of cadmium, 3.2–4.5 mg kg^−1^ of lead, 3.2–4.3 mg kg^−1^ of nickel, 0.83–2.76 mg kg^−1^ of copper, 3.5–17 mg kg^−1^ of zinc, and 52–126 mg kg^−1^ of iron, thus showing that volcanic activity contributed to the distribution of heavy metals on the regions in Tabasco and Chiapas where we collected the samples of artisanal cheeses [51].

### 2.6. Copper

Copper is an essential microelement active in vital functions of the human body; however, the ingestion of high concentrations of copper might cause health issues [49]. The results of our analyses of cheese samples made in Tabasco and Chiapas showed copper concentrations below the maximum limits established by the FAO/WHO [4]. The average, minimum, and maximum copper concentrations in mg kg^−1^ that we observed in cheese samples from Tabasco were 0.0199 ± 0.021, 0.0059, and 0.0437. By cheese variety, the average, minimum, and maximum copper concentrations in mg kg^−1^ were 0.0285, 0.0129, and 0.0402 for crema, 0.0233, 0.0059, and 0.0884 for oaxaca, 0.0093, 0.0078, and 0.0108 for fresco, and 0.0148, 0.0059, and 0.0346 for panela. In the cheese samples from Chiapas, the average, minimum, and maximum copper concentrations in mg kg^−1^ that we recorded were 0.0202 ± 0.022, 0.0007, and 0.0354. By cheese variety, the average, minimum, and maximum copper concentrations in mg kg^−1^ were as follows: 0.0271, 0.0167, and 0.0354 for crema; 0.0141, 0.0067, and 0.0223 for oaxaca; 0.0267, 0.0113, and 0.0337 for cotija; and 0.0165, 0.0059, and 0.0285 for panela. No significant differences in copper concentrations were found between the artisanal cheeses from Tabasco and Chiapas. The paired data test was found to be TC = +0.3578, a value within the CV range (−2.672 to +2.672) that was confirmed by the Tukey’s test (Table 5d).

**Figure 4:**
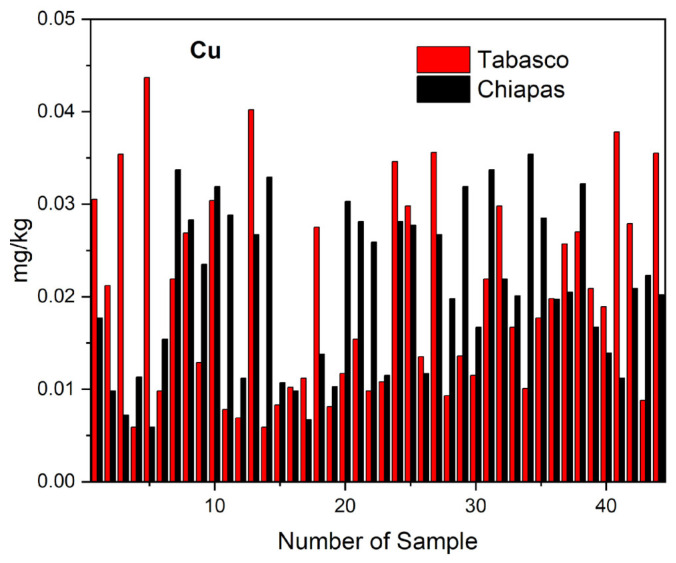
Distribution of copper in the different varieties of artisanal cheeses from Tabasco and Chiapas.

The distribution of copper concentrations we observed in the cheese samples from Tabasco and Chiapas shown in Figure 4 shows a high variation in such concentrations. By cheese variety, our results determined the samples of crema contained copper at concentrations ranging between 0.0402 and 0.0129 mg kg^−1^, of oaxaca between 0.0437 and 0.0007 mg kg^−1^, of cotija between 0.0337 and 0.0113 mg kg^−1^, of frescos between 0.0197 and 0.0078 mg kg^−1^, and of panela between 0.0346 and 0.0039 mg kg^−1^. We can attribute this variability and the low concentration of copper in the cheese samples from Tabasco and Chiapas to the states’ soil type variation. In both states the soil types include Gleysol, Fluvisol, Histosol, Leptosol-Vertisol, Acrisol, Vertisol, Luvisol, and Ferralsol, whose differences in the values of pH, electric conductivity, cation exchange capacity, and organic matter content are factors determining the amount of extractable concentration of copper in each soil type, in studies carried out by Palma and the National Institute of Statistic and Geography [52,53]. Studies carried out by De la Cruz report extractable copper concentrations of 0.99–2.45 mg kg^−1^ in Vertisols and 0.98–2.45 mg kg^−1^ in Fluvisoles, and Salgado reported extractable copper concentrations in subunits of Acrisols of 0.60 ± 0.30 mg kg^−1^ (subunit ACdyhfr) 0.80 ± 0.50 mg kg^−1^ (ACfrpl), 0.40 ± 0.30 mg kg^−1^ (ACfrum), 1.10 ± 0.20 mg kg^−1^ (ACglpl), 0.80 ± 0.50 mg kg^−1^ (AChupl), 0.60 ± 0.50 mg kg^−1^ (AChuum), 0.80 ± 0.20 mg kg^−1^ (ACumgl) and 0.70 ± 0.50 mg kg^−1^ (ACumpl) [35,36]. The value of copper concentration that we found in the analyzed cheese samples was below the 0.40 mg L^−1^ maximum permitted limit in milk and dairy products by the European Commission (EC) [54]. The copper contained in the artisanal cheese samples from Tabasco and Chiapas may contribute to the recommended daily ingestion of 3 mg for adults FAO/WHO [4].

Oaxaca and ranchero cheeses made in Santa Ana Xalmimilulco, Puebla, Castro reported a 0.02 mg kg^−1^ copper content, and for cheese samples from markets in the city of Puebla [55]. In Egypt, Meshref reported copper concentrations of between 0.002 and 0.53 mg kg^−1^ in samples of Kareish cheese made in the Beni-Suef region [22]. In Europe, Elbarbary and Hamouda reported copper concentrations of 3.25 ± 1.06 mg kg^−1^ for feta cheese [56], and Reinholds found an average copper concentration of 0.29 mg kg^−1^ in cheeses made in the Kvemo Kartli region of Georgia [14]. Al Sidawi determined an average copper concentration of 1.261 ± 0.739 mg kg^−1^ in imeruli cheese and 2.463 ± 2.314 mg kg^−1^ in sulguni cheese [49]. Previous reports of copper content in cheese samples are similar to or higher than those we determined in our analysis of cheese varieties made in the states of Tabasco and Chiapas.

### 2.7. Zinc

Zinc is another essential element that when ingested at high concentrations might lead to neurological, hematological, immunological, renal, hepatic, cardiovascular, and genotoxic conditions [23]. The average, minimum, and maximum concentrations of zinc in mg kg^−1^ that we determined in the cheese samples from the states of Tabasco were 0.161 ± 0.18, 0.0204, and 0.301 mg kg^−1^. By cheese variety, the average, minimum, and maximum zinc concentrations in mg kg^−1^ we observed were as follows: 0.0874, 0.0204, and 0.2418 for crema; 0.1579, 0.0317, and 0.2645 for oaxaca; 0.2584, 0.2435, and 0.2785 for fresco; and 0.2437, 0.1436, and 0.2927 for panela. For the samples from Chiapas, the average, minimum, and maximum zinc concentrations in mg kg^−1^ that we observed were 0.194 ± 0.21, 0.0421, and 0.437 mg kg^−1^. By cheese variety, the average, minimum, and maximum zinc concentrations in mg kg^−1^ we recorded were as follows: 0.1008, 0.0421, and 0.3699 for crema; 0.2437, 0.0677, and 0.4369 for oaxaca; 0.2216, 0.1664, and 0.3708 for cotija; and 0.2181, 0.1325, and 0.3308 for panela. It was found that there are no significant differences in the Zn concentrations in the Tabasco and Chiapas cheese samples. In Table 5e, it is observed that Tukey’s test presents only one grouping (A) and the Student’s *t*-test with TC (−1.376) is between the critical values VC (−2.672; +2.672).

**Figure 5:**
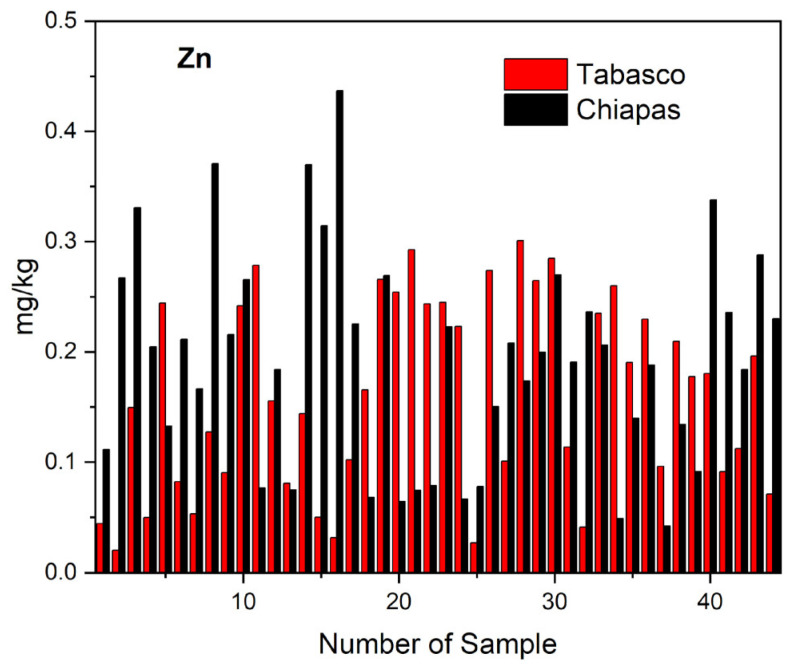
Distribution of zinc in the different varieties of artisanal cheeses from Tabasco and Chiapas.

As seen in Figure 5, we found an ample variation in the zinc concentration of the cheese samples that we analyzed, for example, these values (expressed in mg kg^−1^) were 0.0204–0.369 for crema, 0.032–0.437 for oaxaca, 0.188–0.278 for cotija, 0.167–0.374 for fresco, and 0.132–0.334 for panela. Such variability in zinc content might have been due to the diversity of soil types in Tabasco and Chiapas, and to the physicochemical variables of these types [52], which agrees both with the similarities in soil types between both states and with the recognition that the content of zinc in the cheese samples was due to the soil types present in the geographic regions where they were collected, as previously reported in other studies. For example, De la Cruz determined Zn concentrations of 0.68–0.85 mg kg^−1^ in Fluvisols, and of 0.66–0.87 mg kg^−1^ in Vertisols, and in different Acrisol units [35], and Salgado reported Zn concentrations in mg kg^−1^ of 0.30 ± 0.20 (ACdyhfr), 0.50 ± 0.30 (ACfrpl), 0.40 ± 0.10 (ACfrum), 0.50 ± 0.20 (ACglpl), 0.60 ± 0.50 (AChupl), 0.30 ± 0.20 (AChuum), 0.30 ± 0.20 (ACumgl), and 0.51 ± 0.30 (ACumpl) [36].

The recommended daily intake of zinc for adult women and men is 8 mg and 11 mg kg^−1^, respectively. The range of zinc concentration we found in cheese samples from Tabasco and Chiapas suggests that their consumption might contribute to satisfy the minimum requirement of zinc for humans. Our results agree with those of Castro for cheeses in the Mexican state of Puebla, who reported zinc concentrations of 0.18 ± 0.09 mg kg^−1^ for oaxaca and 0.74 ± 0.1 mg kg^−1^ for ranchero cheeses [17]. In work carried out by Ghafari and Sobhanardakani reported 0.198 mg kg^-1^ of zinc in the cheeses produced from Hamedan, Iran [57]. Al Sidawi reported zinc contents of 75.86 ± 52.528 mg kg^−1^ in imeruli and 124.8 ± 97.775 mg kg^−1^ in sulguni cheeses manufactured in the province Kartli, Georgia [49]. For blue cheese sold in the European Union, Reinholds reported zinc concentrations between 10.4 and 39.5 mg kg^−1^ [14]. For different cheeses made in Turkey, Mendil and Orak reported 12.0 mg kg^−1^ and 15.57 mg kg^−1^ of zinc for white cheese [58,59]; Çetinkaya and collaborators found an average zinc concentration of 27.52 ± 1.85 mg kg^−1^ in cheese samples in the Cami Bogazi region and for white cheeses [24]. The authors attributed the presence of zinc in cheeses to the use of contaminated machinery and containers during cheese making, and to the transport of zinc through the food web due to the environmental pollution of soil, water, and fodder.

### 2.8. Iron 

**Figure 6:**
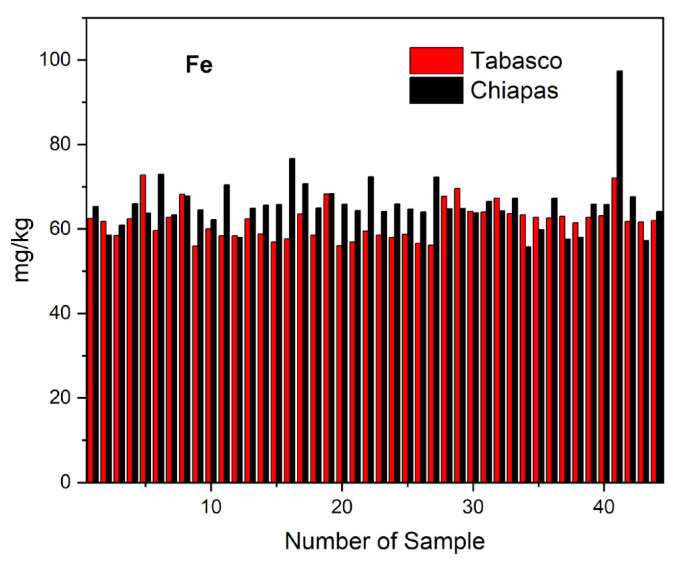
Distribution of iron in the different varieties of artisanal cheeses from Tabasco and Chiapas.

Iron is essential for oxygen transportation and storage in the human body, but ingestion of large quantities of iron may lead to blood, heart, kidney, and endocrine system conditions, cellular damage, and mutations [25,60]. Figure 6 shows the distribution of Fe in the cheese samples from Tabasco and Chiapas.

The average, minimum, and maximum Fe concentrations in mg kg^−1^ that we found in cheese samples from Tabasco were 61.84 ± 4.23, 55.97, and 72.76, whereas for the samples from Chiapas were 62.58, 55.97, and 72.08 for crema, 62.15, 56.92, and 72.76 for oaxaca, 61.61, 58.38, and 68.29 for fresco, and 60.25, 56.07, and 67.77 for panela. For cheese samples from Chiapas, the average, minimum, and maximum Fe concentrations in mg kg^−1^ were 65.76 ± 6.61, 55.82, and 97.41, and by cheeses variety were as follows: 64.37, 55.82, and 72.33 for crema; 68.09, 57.96, and 97.41 for oaxaca; 65.91, 62.19, and 72.09 for cotija; and 64.72, 59.86, and 72.94 for panela. The results of the Fe concentrations of Tabasco and Chiapas cheeses showed significant differences since the paired Student’s *t*-test gave a value of TC = −3.696, which is outside the range of CV (−2.672 to +2.672). The Tukey’s test showed two groupings (A) and (B), thus confirming the previous results (Table 5f).

We attributed the presence of Fe in the analyzed cheese samples from Tabasco and Chiapas to soil types in the region, where Ferralsols rich in iron sesquioxides are dominant. For example, the Acrisols—soils which are easily identified by their yellowish to reddish dark color, strong acidity, and a B horizon accumulating alluvial clay—are rich in iron and aluminum sesquioxides. Salgado and Palma analyzed Fe concentrations in subunits of Acrisols finding 67.00 ± 52.00 (ACdyhfr), 76.00 ± 21.00 (Acfrpl), 51.00 ± 16.00 (Acfrum), 115.00 ± 58.00 (Acglpl), 85.00 ± 29.00 (Achupl), 94.00 ± 29.00 (Achuum), 72.00 ± 15.00 (Acumgl), and 64.00 ± 49.00 (Acumpl) mg kg^−1^ [36,52]. Additionally, De la Cruz found Fe concentrations of 67.6 in Vertisols and 112.2 mg kg^−1^ in Fluvisols [35]. The above-mentioned soil types are rich in organic matter with high concentrations of humic and fulvic acids, which give soil acidic pH values between 4.6 and 5.7, chemical conditions that facilitate the biosorption of iron by grass rhizomes and its accumulation in stalks.

According to our results, the concentrations of iron that we observed in cheese samples from Tabasco and Chiapas are within the parameters for human consumption and may cover a large part of the recommended daily intake of 8 mg for adult males, 18 mg for adult females, 11 mg for young males, 15 mg for young females, and 27 mg for pregnant women. Our results were similar to those of Al Sidawi who analyzed cheese samples from the Kvemo Kartli (Georgia), finding Fe concentrations of 69.09 ± 64.918 mg kg^−1^ in imeruli and 101.1 ± 91.166 mg kg^−1^ in sulguni cheeses [49]. Jalili also found Fe concentrations between 67.7 ± 4.5 and 71.3 ± 4.9 mg kg^−1^ in iron-fortified feta cheeses [61]. In contrast, the values for Fe concentration we found exceed the values reported in previous studies made in Turkey by Kirdar in akcaka tik cheeses between 7.49 and 29.05 mg kg^−1^ [62]. While Centinkaya in cami bogazi cheeses from Trabzon (Turkey) reported 0.371 ± 0.177 mg kg^−1^ Fe. Meshref found Fe concentrations between 1.763 and 17.739 ppm in kareish cheeses from the Beni-Suef region [22], and in Europe, Reinholds reported Fe concentrations between 1.57 and 12.4 mg kg^−1^ in blue cheeses [14].

### 2.9. Risk Analysis

**Figure 7:**
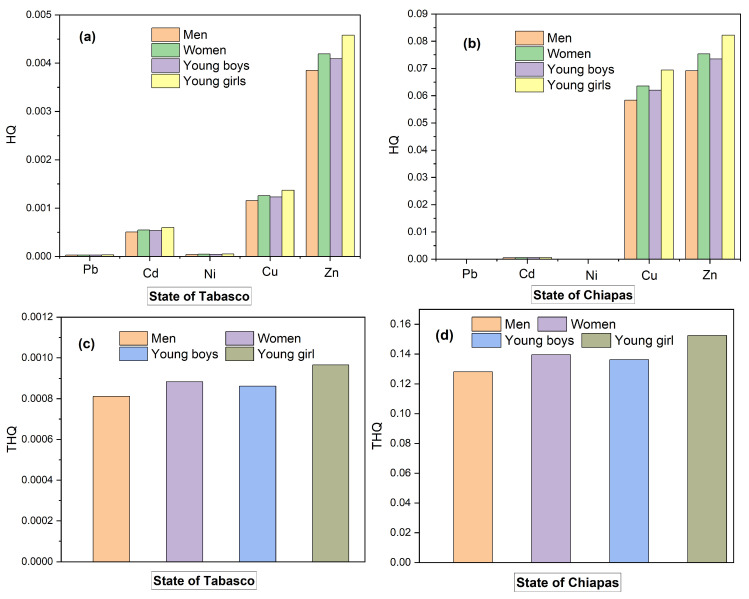
Values of the risk index and contribution of each metal for adults and youngsters (**a**,**b**); values of the total risk index for adults and youngsters (**c**,**d**).

The values of *HQ* we estimated for the concentrations of the heavy metals lead, cadmium, nickel, copper, and zinc that we determined in cheese samples from Tabasco are shown in Figure 7a,b for the samples from Chiapas. Zinc, copper, and cadmium were the metals that contributed the most to the *HQ* values of samples from Tabasco in the order young girls > adult woman > young boys > adult man (Figure 7a), while for cheese samples from Chiapas, zinc and copper were the metals having the highest contribution to the *HQ* values in the same order as for Tabasco. However, all the *THQ* values that we estimated were lower than one (Figure 7c,d), meaning that there is no risk of developing medical conditions from intake of heavy metals contained in the cheese varieties we analyzed.

In previous studies, Castro reported values of *THQ* below one for arsenic in milk samples from the upper Balsas River region in the state of Puebla [17,21]; Reinholds found *THQ* between 0.05 and 0.14 for youngsters, and between 0.03 and 0.09 for adults for the intake of blue cheese consumed by the populations from Denmark, France, Italy, Spain, and the UK [14], and Zafarzadeh reported *THQ* values of 1.11, 1.33, and 5.42 for adults and youngsters due to the intake of cadmium-polluted butter from the Gorgan region in Iran [63], which poses a high health risk for the inhabitants of that geographic area.

Table 6 shows the results of the Tukey tests for CR and CRT considering the variables heavy metals (Pb, Cd, and Ni) and age group (youngsters and adults). The results revealed significant differences in the effect on CR of each metal in the order Cd > Pb > Ni, which means that the ingestion by youngsters and adults of even low concentrations of Cd could represent a high *CR* relative to the ingestion of Pb or Ni; however, the *CR* will depend on the consumed cheese variety. The significant differences in *CR* between age and weight groups (Table 6a,b) could have been due to adults having slower metabolic rates than youngsters [21]. Table 6c shows no significant differences in *CRT* between adults and youngsters, possibly due to the low contents of Pb, Cd, and Ni of the artisanal cheeses made in Tabasco and Chiapas.

**Figure 8:**
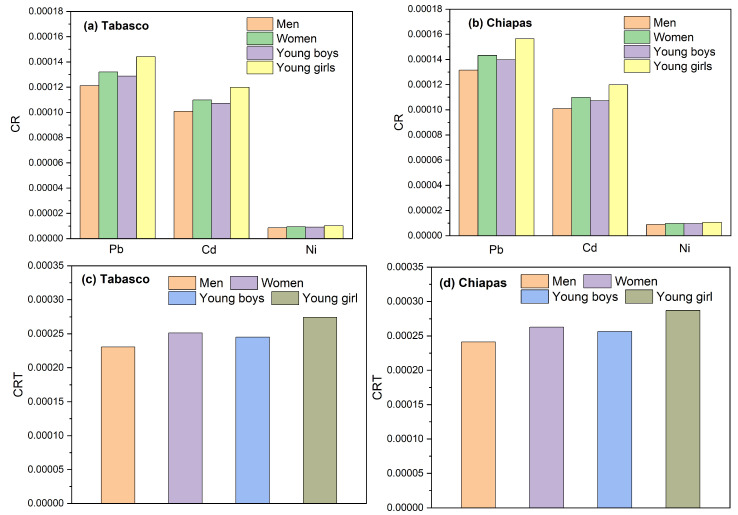
Values of the cancer risk index and contribution of each metal, for adults and youngsters (**a**,**b**); values of the *CRT* index for adults and youngsters (**c**,**d**).

The *CR* and *CRT* values we estimated for adults and youngsters are shown in Figure 8a,b. In our study, lead and cadmium were the heavy metals that contributed the most to cancer risk in both states, with a higher value for women than for men. The values of *CRT* were in the decreasing order of young girls > adult women > young boys > adult men. In general, as seen in Figure 8c,d, all the *CRT* values we estimated were within the range of 10^−4^–10^−6^ established by the US-EPA [8].

Our *CRT* estimates were lower than the values of 0.0018–0.014 reported by Castro for children and youngsters in the state of Puebla from the intake of milk containing heavy metals due to contamination of fodder irrigated with the polluted water from the Atoyac, Xochiac, and Xopanac rivers [21].

## 3. Materials and Methods

### 3.1. Artisanal Cheese Samples

**Figure 9:**
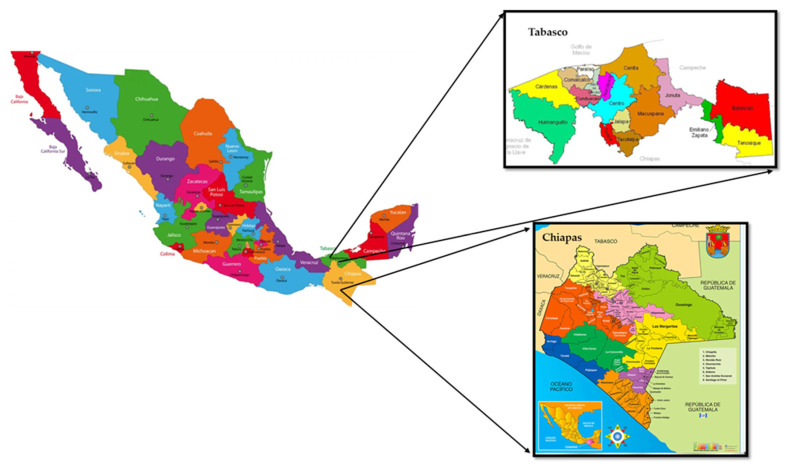
Municipalities of Tabasco and Chiapas, where samples of artisanal cheese were collected.

**Figure 10:**
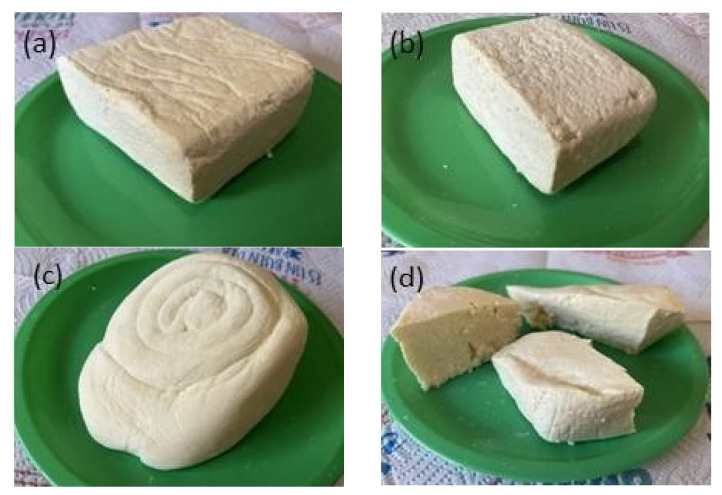

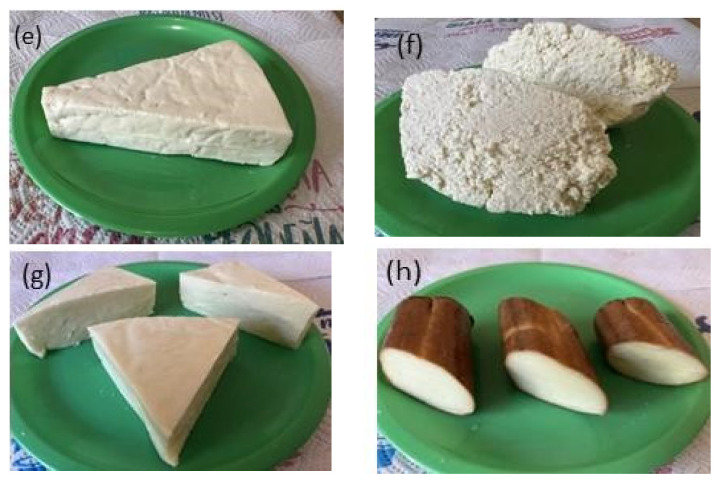
Artisanal cheeses from Tabasco and Chiapas: (**a**) double cream, (**b**) cream or soup, (**c**) oaxaca, (**d**) panela, (**e**) cotija, (**f**) fresco, (**g**) mozzarella, (**h**) provolone.

For the analysis of heavy metals, we acquired 44 samples of artisanal cheeses from seven varieties (doble crema, crema, oaxaca, fresco, panela, mozzarella, and provolone) in four municipalities (Huimanguillo, Balancán, Tenosique, and Centro) of the state of Tabasco, and 44 samples from five varieties (doble crema, oaxaca, panela, cotija, and fresco) in four municipalities (Solosuchiapa, Juárez, Catazajá, and Reforma) of the state of Chiapas. The average weight of samples was 1 kg (Figure 9 and Figure 10).

### 3.2. Sample Preparation

All the glassware used in the experimental procedures was washed in a 5% hydrochloric acid solution for 24 h and afterward rinsed with deionized water to remove possible contaminants that could interfere with the results of the analyses. Cheese samples were digested following the method of [19]. Samples were first homogenized by being finely chopped (approximately 2 mm). A 1 g aliquot of each sample homogenate was weighted in an Ohaus analytical balance and placed in a porcelain crucible and calcined in a Thermo Scientific-BF51794C-1 furnace at 450–500 °C for 16 h and, after, slowly cooled to room temperature. The resulting ashes were treated with 1 mL of concentrated nitric acid and calcined once more at 450–500 °C for 6 h to destroy the organic matrix. After the second calcination, samples were dissolved in 2 mL of concentrated nitric acid and 2 mL of hydrogen peroxide, after which the resulting solutions were filtered with no. 41 Whatman paper and the filtrates were diluted to 100 mL in a 100 mL volumetric flask. Each final solution was placed in a Teflon jar and stored at 10 °C until they were analyzed in a Thermo Scientific ICE 3000 Series atomic absorption spectrometer as described below.

### 3.3. Analytical Parameters for Atomic Absorption Spectrometry

To determine the concentration in samples of heavy metals (measured in mg L^−1^), calibration curves were run at different wavelengths for each heavy metal analyzed as follows: 217.0 nm for Pb, 228.8 nm for Cd, 232.0 nm for Ni, 324.8 nm for Cu, 213.9 nm for Zn, and 248.3 nm for Fe. A mixture of air–acetylene combustion gas was used and the wavelength was adjusted for each heavy metal.

### 3.4. Risk Analysis

#### 3.4.1. Daily Intake of Metals

The health risks of human intake of artisanal cheeses made in Tabasco and Chiapas were estimated based on the results of heavy metal determinations in the analyzed samples by assuming a per capita yearly intake of 6 kg—taking into account the yearly intake value of between 4 and 8 kg established for developing countries by the OECD/FAO [12]—using Equation (1) as suggested by Castro [21].

(1) $$Chronic\;Daily\;Intake\;CDI=\frac{\left(C\;metal\right)\left(D\;intake\right)}{B\;average\;weight}\;\;\;$$

where *C metal* is the metal concentration determined in cheese samples in mg kg^−1^, *D intake* is the per capita consumption of cheese in kg yr^−1^), and *B average weight* is the body weight of individuals in kg.

#### 3.4.2. Hazard Quotient

The hazard quotient was estimated by Equation (2).

(2) $$HQ=\frac{CDI}{RfD}\;\;\;$$

where *HQ* is the hazard quotient, CDI is chronic daily intake, *RfD* is oral reference dose value of exposure to the chronic daily intake in mg kg^−1^. Table 7 contains the values used for the calculation of *HQ* [21]. 

#### 3.4.3. Total Hazard Quotient

The total hazard quotient is calculated by adding the *HQ* values for each metal (Equation (3)).

(3) $$THQ=\sum \left(HQ_{Cd}+HQ_{Pb}+HQ_{Ni}+HQ_{Cu}+HQ_{Zn}+HQ_{Fe}\right)$$

where *THQ* is the total hazard quotient, *HQ_Cd_* is the hazard quotient for cadmium, *HQ_Pb_* is the hazard quotient for lead, *HQ_Ni_* is the hazard quotient for nickel, *HQ_Cu_* is the hazard quotient for copper, *HQ_Zn_* is the hazard quotient for zinc, and *HQ_Fe_* is the hazard quotient for iron. According to the recommendations of the US-EPA [8], values of *THQ* smaller than one imply no risk, while values of *THQ* greater than one suggest a high risk for human health [21].

#### 3.4.4. Cancer Risk and Total Cancer Risk

The total cancer risk was calculated by adding the cancer risk values for the carcinogenic metals cadmium, nickel, and lead (Equations (4) and (5)) recommended by Reinholds [14].

(4) $$CR=\frac{CDI}{Sf}\;\;\;\;$$

(5) $$CRT=\sum CR_{Pb+Cd+Ni}$$

where *CR* is the cancer risk, *CRT* is the total cancer risk, *CR_Cd+Ni+Pb_* is the cancer risk for cadmium, nickel, lead, *CDI* is chronic daily intake, and slope factors (*Sf*). The average body weights of the Mexican population were established by the OECD/FAOto be 74.8 kg for adult males, 68.7 kg for adult females, 62.9 kg for young females, and 70.4 kg for young males [12]. 

## 4. Conclusions

The contents of Pb, Cd, Ni, Cu, and Zn showed no significant differences between cheese samples from the states of Tabasco and Chiapas. The concentrations of Pb, Cd, and Ni that we determined in all cheese samples were below the values established by the *Codex Alimentarius* and FAO/WHO. The heavy metals Zn, Cu, and Ni contributed the most to the *HQ* values. All *THQ* values were smaller than one, which implies no hazard from the intake of the cheese varieties we analyzed and were in the descending order young females > adult females > young males > adult males. The heavy metals Pb and Cd contributed the most to the *CR* and *CRT* values—which followed the descending order young girls > adult females > young boys > adult males—that were within the values established by the US-EPA, therefore indicating no cancer risk due to the intake of the analyzed cheeses. Considering the population growth and increased anthropic activities in Tabasco and Chiapas, it is essential to determine the presence in locally made cheeses of other carcinogenic heavy metals like arsenic, mercury, chromium, and vanadium for their incorporation into the estimations of the *THQ* and *CRT* values. We attributed the concentrations of Cu, Zn, and Fe in the analyzed cheese samples to the soil types present in Tabasco and Chiapas.

## Figures and Tables

**Table 1:** Metal concentrations in mg kg^−1^ in samples of different varieties of artisan cheeses made in the state of Tabasco, Mexico.

Cheese Variety	Municipality	Pb	Cd	Ni	Cu	Zn	Fe
double cream	Tenosique	0.0047	0.0027	0.0002	0.0305	0.0443	62.48
double cream	Tenosique	0.0040	0.0021	0.0054	0.0212	0.0204	61.79
oaxaca	Tenosique	0.0035	0.0000	0.0024	0.0354	0.1494	58.48
oaxaca	Tenosique	0.0047	0.0004	0.0048	0.0059	0.0496	62.41
oaxaca	Tenosique	0.0028	0.0001	0.0032	0.0437	0.2443	72.76
oaxaca	Balancán	0.0029	0.0015	0.0005	0.0098	0.0823	59.65
cream or soup	Balancán	0.0046	0.0031	0.0096	0.0219	0.0532	62.73
double cream	Balancán	0.0043	0.0019	0.0037	0.0269	0.1271	68.26
double cream	Balancán	0.0057	0.0037	0.0012	0.0129	0.0904	55.97
cream or soup	Balancán	0.0078	0.0049	0.0018	0.0304	0.2418	60.04
fresh	Balancán	0.0068	0.0037	0.0042	0.0078	0.2785	58.38
oaxaca	Balancán	0.0041	0.0001	0.0041	0.0069	0.1554	58.39
cream or soup	Centro	0.0092	0.0063	0.0078	0.0402	0.0808	62.39
panela	Centro	0.0043	0.0019	0.0004	0.0059	0.1436	58.82
oaxaca	Centro	0.0044	0.0031	0.0005	0.0083	0.0499	56.92
oaxaca	Centro	0.0036	0.0016	0.0011	0.0102	0.0317	57.67
mozzarella	Huimanguillo	0.0035	0.0026	0.0007	0.0112	0.1019	63.58
provolone	Huimanguillo	0.0048	0.0001	0.0015	0.0275	0.1653	58.52
fresh	Huimanguillo	0.0034	0.0002	0.0033	0.0081	0.2659	68.29
panela	Huimanguillo	0.0043	0.0075	0.0019	0.0117	0.2542	56.07
panela	Huimanguillo	0.0039	0.0013	0.0009	0.0154	0.2927	56.93
fresh	Huimanguillo	0.0005	0.0004	0.0022	0.0098	0.2435	59.46
fresh	Huimanguillo	0.0041	0.0002	0.0021	0.0108	0.2447	58.56
panela	Huimanguillo	0.0047	0.0021	0.0034	0.0346	0.2231	57.98
double cream	Huimanguillo	0.0004	0.0047	0.0048	0.0298	0.0271	58.72
panela	Huimanguillo	0.0044	0.0001	0.0038	0.0135	0.2738	56.61
double cream	Huimanguillo	0.0038	0.0027	0.0069	0.0356	0.1008	56.18
panela	Huimanguillo	0.0031	0.0011	0.0027	0.0093	0.3008	67.77
oaxaca	Huimanguillo	0.0043	0.0019	0.0042	0.0136	0.2645	69.53
panela	Huimanguillo	0.0039	0.0003	0.0056	0.0115	0.2842	64.21
cream or soup	Huimanguillo	0.0063	0.0027	0.0087	0.0219	0.1135	63.98
double cream	Huimanguillo	0.0039	0.0035	0.0092	0.0298	0.0413	67.28
panela	Huimanguillo	0.0042	0.0009	0.0052	0.0167	0.2348	63.61
fresh	Huimanguillo	0.0041	0.0006	0.0034	0.0101	0.2598	63.34
oaxaca	Huimanguillo	0.0037	0.0009	0.0023	0.0177	0.1902	62.79
oaxaca	Huimanguillo	0.0064	0.0000	0.0015	0.0198	0.2295	62.56
cream or soup	Huimanguillo	0.0044	0.0026	0.0092	0.0257	0.0961	63.03
oaxaca	Huimanguillo	0.0084	0.0007	0.0039	0.0271	0.2095	61.47
oaxaca	Huimanguillo	0.0051	0.0000	0.0049	0.0209	0.1774	62.78
oaxaca	Huimanguillo	0.0043	0.0014	0.0044	0.0189	0.1805	63.15
double cream	Huimanguillo	0.0052	0.0156	0.0132	0.0378	0.0912	72.08
cream or soup	Huimanguillo	0.0125	0.0039	0.0032	0.0279	0.1121	61.77
oaxaca	Huimanguillo	0.0049	0.0011	0.0029	0.0088	0.1962	61.63
double cream	Huimanguillo	0.0072	0.0084	0.0042	0.0355	0.0708	62.04
	Average	0.0047 ± 0.002	0.0023 ± 0.002	0.0039 ± 0.004	0.0199 ± 0.02	0.1611 ± 0.18	61.84 ± 4.23
	Maximum	0.0125	0.0056	0.0132	0.0437	0.3008	72.76
	minimum	0.0004	0	0.0002	0.0059	0.0204	55.97

**Table 2:** Metal concentrations in mg kg^−1^ in samples of different varieties of artisan cheeses made in the state of Chiapas, Mexico.

Cheese Variety	Municipality	Pb	Cd	Ni	Cu	Zn	Fe
double cream	Solosuchiapa	0.0029	0.0041	0.0063	0.0177	0.1114	65.34
oaxaca	Solosuchiapa	0.0081	0.0000	0.0019	0.0098	0.2669	58.52
panela	Solosuchiapa	0.0007	0.0003	0.0012	0.0072	0.3308	60.89
cotija	Solosuchiapa	0.0047	0.0051	0.0009	0.0113	0.2047	65.98
panela	Solosuchiapa	0.0078	0.0034	0.0017	0.0059	0.1325	63.75
panela	Solosuchiapa	0.0067	0.0013	0.0031	0.0154	0.2112	72.94
cotija	Solosuchiapa	0.0056	0.0043	0.0043	0.0337	0.1664	63.33
cotija	Juárez	0.0047	0.0038	0.0075	0.0283	0.3708	67.89
cotija	Juárez	0.0039	0.0037	0.0056	0.0235	0.2157	64.52
cotija	Juárez	0.0049	0.0062	0.0037	0.0319	0.2654	62.19
cream or soup	Juárez	0.0051	0.0055	0.0091	0.0288	0.0766	70.49
oaxaca	Juárez	0.0083	0.0009	0.0016	0.0112	0.1839	57.96
double cream	Juárez	0.0069	0.0077	0.0015	0.0267	0.0748	64.86
double cream	Juárez	0.0035	0.0044	0.0039	0.0329	0.3699	65.65
oaxaca	Juárez	0.0033	0.0017	0.0052	0.0107	0.3145	65.79
oaxaca	Juárez	0.0047	0.0033	0.0044	0.0198	0.4369	76.67
oaxaca	Catazajá	0.0041	0.0013	0.0038	0.0067	0.2254	70.74
oaxaca	Catazajá	0.0003	0.0021	0.0021	0.0138	0.0677	64.91
oaxaca	Catazajá	0.0031	0.0027	0.0029	0.0103	0.2691	68.37
double cream	Catazajá	0.0075	0.0033	0.0078	0.0303	0.0642	65.87
cream or soup	Catazajá	0.0069	0.0031	0.0067	0.0281	0.0742	64.39
double cream	Catazajá	0.0084	0.0037	0.0025	0.0259	0.0787	72.33
oaxaca	Catazajá	0.0041	0.0011	0.0030	0.0155	0.2228	64.15
double cream	Catazajá	0.0069	0.0029	0.0039	0.0281	0.0665	65.94
double cream	Catazajá	0.0053	0.0033	0.0046	0.0277	0.0776	64.68
oaxaca	Catazajá	0.0008	0.0046	0.0026	0.0117	0.1505	64.02
cotija	Catazajá	0.0088	0.0047	0.0079	0.0267	0.2081	72.09
cotija	Catazajá	0.0045	0.0032	0.0061	0.0198	0.1735	64.75
cotija	Catazajá	0.0081	0.0041	0.0044	0.0319	0.1996	65.82
panela	Catazajá	0.0005	0.0007	0.0025	0.0167	0.2697	63.81
cotija	Catazajá	0.0041	0.0046	0.0027	0.0337	0.1908	66.58
panela	Catazajá	0.0011	0.0002	0.0006	0.0219	0.2364	64.33
panela	Catazajá	0.0009	0.0001	0.0019	0.0201	0.2062	67.51
double cream	Reforma	0.0067	0.0049	0.0054	0.0354	0.0489	55.82
panela	Reforma	0.0022	0.0023	0.0009	0.0285	0.1395	59.86
fresh	Reforma	0.0067	0.0011	0.0003	0.0197	0.1879	67.26
double cream	Reforma	0.0089	0.0051	0.0058	0.0205	0.0421	57.61
cream or soup	Reforma	0.0056	0.0045	0.0041	0.0322	0.1341	58.04
double cream	Reforma	0.0073	0.0026	0.0036	0.0167	0.0912	65.87
oaxaca	Reforma	0.0062	0.0019	0.0005	0.0139	0.3378	65.83
oaxaca	Reforma	0.0033	0.0034	0.0022	0.0112	0.2359	97.41
oaxaca	Reforma	0.0056	0.0008	0.0019	0.0209	0.1832	67.61
oaxaca	Reforma	0.0063	0.0037	0.0035	0.0223	0.2877	57.62
oaxaca	Reforma	0.0034	0.0003	0.0007	0.0202	0.2301	64.13
	Average	0.0051 ± 0.002	0.0023 ± 0.002	0.0031 ± 0.004	0.0202 ± 0.022	0.194 ±0.21	65.76 ± 6.61
	Maximum	0.0089	0.0077	0.0091	0.0354	0.437	97.41
	Minimum	0.0003	0.0000	0.0003	0.0059	0.0421	55.82

**Table 3:** Moisture content in artisanal cheeses from the states of Tabasco and Chiapas.

Sample	Cream	Cotija	Oaxaca	Fresh	Panela
1	42.594	39.295	60.756	58.320	55.610
2	49.741	36.080	64.414	58.327	63.113
3	51.560	39.352	59.361	63.910	60.901
4	41.018	40.690	63.062	62.455	60.318
5	45.862	37.750	61.025	61.325	62.862
6	45.810	41.786	63.942	60.458	64.538
7	37.338	41.949	64.956	-	54.937
8	43.719	34.565	59.746	-	63.710
9	45.081	36.829	60.344	-	65.707
10	44.866	-	63.582	-	62.195
11	46.634	-	62.431	-	65.820
12	35.921	-	60.679	-	66.447
13	46.789	-	61.430	-	55.019
14	39.808	-	61.977	-	61.770
15	47.096	-	55.727	-	56.922
16	48.561	-	54.637	-	-
17	48.647	-	55.183	-	-
18	45.336	-	56.782	-	-
19	48.025	-	66.109	-	-
20	50.020	-	55.631	-	-
21	47.559	-	54.017	-	-
22	49.342	-	62.236	-	-
23	38.601	-	57.339	-	-
24	43.091	-	58.655	-	-
25	47.352	-	56.461	-	-
26	39.398	-	56.894	-	-
27	48.085	-	54.063	-	-
28	49.558	-	55.845	-	-
Average	45.264	38.699	59.546	60.799	61.325
Maximum	51.560	41.949	66.109	63.910	66.447
Minimum	35.921	34.565	54.017	58.320	54.937

**Table 4:** Tukey test to determine significant differences between moisture content in artisanal cheeses from the states of Tabasco and Chiapas. Means with a common letter are not significantly different (*p* > 0.05) α = 0.05.

Cheese Variety	Mean	*n*			
cotija	38.699	9	A		
cream	45.264	28		B	
oaxaca	59.545	28			C
fresh	60.799	6			C
panela	61.324	15			C

**Table 5:** Tukey test to determine significant differences between heavy metal content in artisanal cheeses from the states of Tabasco and Chiapas. Means with a common letter are not significantly different (*p* > 0.05) α = 0.05.

(a) Pb				(b) Cd			
State	Mean	n		State	Mean	n	
Chiapas	0.0047	44	A	Chiapas	0.0024	44	A
Tabasco	0.005	44	A	Tabasco	0.003	44	A
(c) Ni				(d) Cu			
State	Mean	n		State	Mean	n	
Chiapas	0.0036	44	A	Chiapas	0.02	44	A
Tabasco	0.0039	44	A	Tabasco	0.02	44	A
(e) Zn				(f) Fe			
State	Mean	n		State	Mean	n	
Chiapas	0.19	44	A	Chiapas	65.78	44	A
Tabasco	0.16	44	A	Tabasco	61.84	44	B

**Table 6:** Tukey’s tests for significant differences in *CR* and *CRT* by heavy metal and age group. A, B and C letters indicates the significant different between CR and CRT.

(a) *CR*				
Metal	Average			
Ni	9.5 × 10^−6^	A		
Cd	1.1 × 10^−4^		B	
Pb	1.4 × 10^−4^			C
(b) *CR*				
Age group	Average			
Adults	8.2 × 10^−5^	A		
Youngsters	8.9 × 10^−5^		B	
(c) *CRT*				
Age group	Average			
Adults	2.5 × 10^−4^	A		
Youngsters	2.7 × 10^−4^	A		

**Table 7:** Oral reference dose (*RfD*) values and slope factors (*Sf*) of metal considered to be carcinogenic.

Heavy Metal	*RfD* (mg kg^−1^) *	*Sf* (mg kg^−1^ day^−1^) *
Cu *	0.037	-
Ni *	0.02	0.1
Pb **	0.036	0.0085
Cd **	0.001	0.005
Zn *	0.3	-

* US-EPA [8], ** Bermúdez [9].

## Data Availability

Data are contained within the article.
